# Long-term Follow-up of Exstrophy-epispadias Complex from a Lower-middle Income Country: A Case Report and Review of the Literature

**DOI:** 10.7759/cureus.7723

**Published:** 2020-04-18

**Authors:** Omar Irfan, Zoya Fatima Rizwan Ladiwala, Zafar Zaidi

**Affiliations:** 1 Pediatrics, Centre for Global Child Health, Hospital for Sick Children, Toronto, CAN; 2 Medicine, Civil Hospital Karachi, Dow University of Health Sciences, Karachi, PAK; 3 Urology, The Indus Hospital, Indus University of Health Sciences, Karachi, PAK

**Keywords:** exstrophy-epispadias, mitrofanoff, quality of life, bladder augmentation, incontinence

## Abstract

Bladder exstrophy-epispadias complex (EEC) is a rare congenital defect where the abdominal muscles and bones fail to close in the mid-pelvis. It is crucial to understand the health-related quality of life (QOL) of exstrophy patients who have undergone multiple correctional surgeries. We herein discuss a case of bladder EEC that was repaired through a series of procedures at a resource-limited hospital in Karachi, Pakistan.

A 21-year-old male, who was born with EEC, underwent bladder augmentation, Mitrofanoff procedure, bladder neck reconstruction, ureter implantation and a right nephrectomy in a single one-stage procedure during late childhood for urinary incontinence. However, this required a further revision because the urinary incontinence persisted, with difficulty in catheterizing the Mitrofanoff channel. On follow-up after 10 years, our patient currently describes normal QOL with near-normal sexual function. Validated questionnaires for QOL, erectile dysfunction, incontinence and prostatic function were used to assess the patient’s post-operative status in these domains. Through our report, we conclude that such patients can have a normal QOL by means of a holistic multidisciplinary management, which includes timely surgical corrections along with an additional focus on the psycho-social and sexual aspects of this condition.

## Introduction

Exstrophy-epispadias complex (EEC) is a rare (incidence of 2.15/100,000 live births) congenital defect where a child’s abdominal muscles and bones fail to close in the midline, thus exposing the bladder to the environment [1]. It is crucial to understand the health-related quality of life (QOL) of exstrophy patients who have undergone multiple correctional surgeries at an early age. At present, there are relatively a limited number of studies that utilize methods to assess the QOL in such patients. The series of surgical reconstructions can include closure of the bony pelvis, anterior abdominal wall and the bladder followed by correction of epispadias, augmentation of the bladder and eventual creation of a urinary conduit. Albeit existing surgical techniques have acceptable quality of outcomes in terms of preserved renal function, continence and cosmesis, there are still risks of recognized orthopedic and urological complications following reconstructive surgery in patients with EEC [2,3]. Previously from Pakistan, 10-year, 8-year and 7-year institutional surveys from teaching hospitals in Peshawar, Islamabad and Karachi reported a series of 12, 29 and 13 cases, respectively [4-6]. The purpose of this report is to examine the management and QOL of a patient surgically managed for EEC in a resource-limited setting. Our report can be a valuable addition to the medical literature as it sheds light on a chronic yet correctable condition. This will subsequently help create awareness about the importance of a holistic approach in managing this condition, especially among patients as well as young physicians and surgeons.

## Case presentation

A 21-year-old male, resident of a rural part of Pakistan, presented at a tertiary-care hospital in Karachi for follow-up following urogenital reconstruction surgery. Our patient was born prematurely at 34 weeks of gestation with EEC. Shortly after birth, exstrophy was closed with bilateral osteotomies alongside a repair of epispadias.

**Figure 1:**
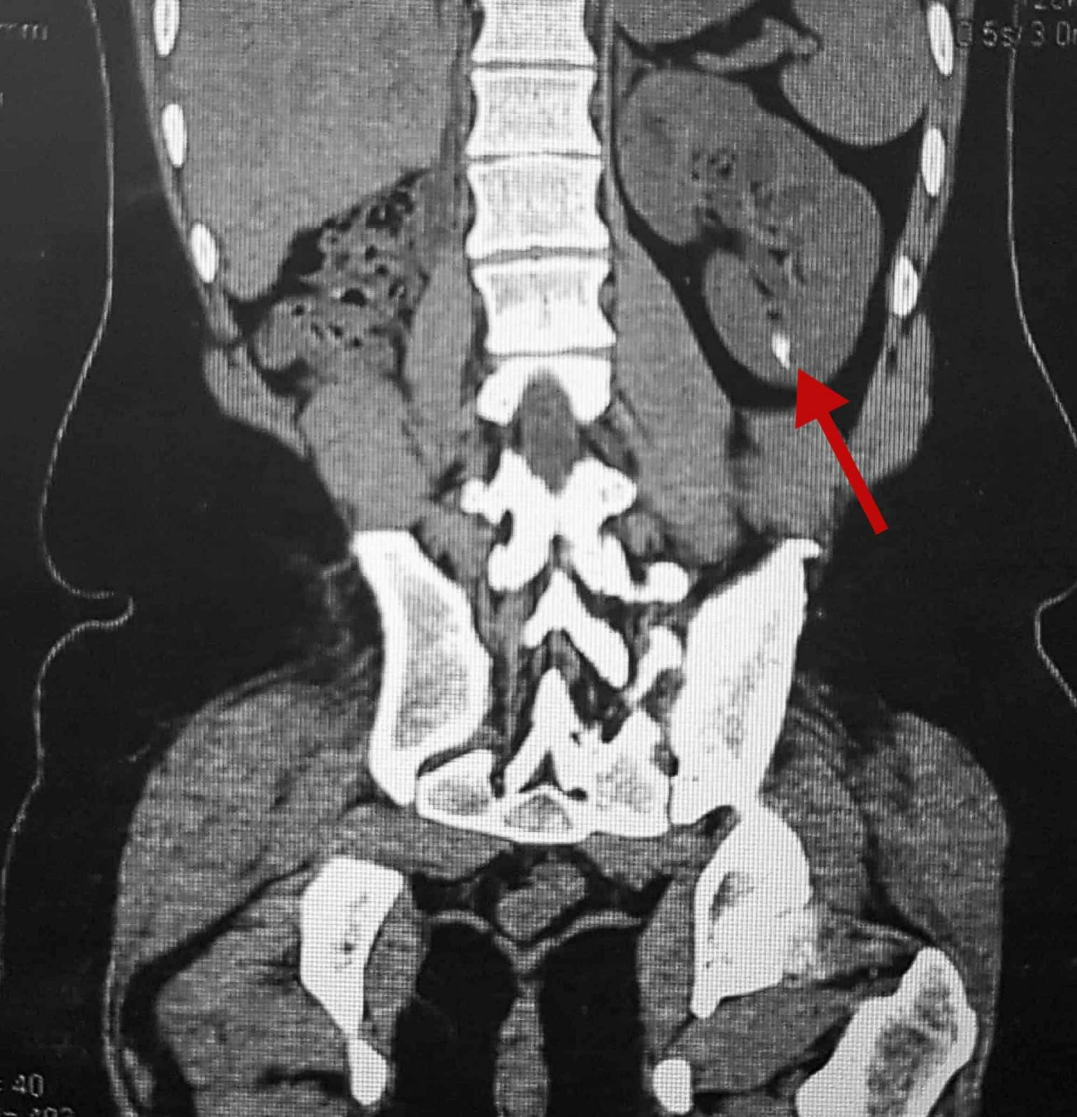
CT scan of the kidney, ureter and bladder showing a small calculus projecting over the lower pole calyx of the left kidney

Our patient suffered from urinary incontinence for the first 11 years of his life, following which he underwent bladder augmentation (ileocystoplasty), Mitrofanoff procedure (appendicovesicostomy with double tube Monti), Young-Dees bladder neck reconstruction with neck closure, ureter implantation and a right nephrectomy in a single one-stage procedure lasting six hours. Up until the surgery, the patient had worn “special need” diapers and underwear to overcome the urinary incontinence. The right kidney, functioning at less than 10%, was scarred and shrunken, and was subsequently removed alongside the right ureter during the surgery. The possible causes of scarring of the right kidney were hydronephrosis and repeated urinary tract infections. Two weeks post-operatively, the procedure required a revision as urinary incontinence persisted with difficulty in catheterizing the Mitrofanoff channel. This was indeed a very distressing time for the patient as well as the family. A post-operative CT scan of the kidney, ureter and bladder also showed a small calculus projecting over the lower pole calyx of the left kidney (Figure 1). No other bowel or bony abnormalities were seen. This was then managed with lithotripsy followed by cystoscopy to clear the bladder fragments; however, a second episode of renal calculus soon followed. 

To date, after a 10-year post-operative period, our patient has a well-healed abdominal incision and the stoma appears healthy and clean. The penis is short with depression of the skin beneath the suprapubic fat pad, and corporal bodies are palpable with a well-shaped glans. The baseline serum creatinine level is between 1.2 and 1.4 mg/dl (normal levels: 0.6-1.2 mg/dl). He can easily catheterize with a 12-Fr suction catheter every two to three hours with a maximum bladder capacity of 500 to 600 ml. The patient regularly irrigates the bladder using normal saline to avoid any mucus accumulation. Surprisingly, our patient describes normal sensation of erection and ejaculation with a near normal semen analysis. Due to catheterization with improper sterilization techniques, the patient develops urinary tract infections (UTIs) from time to time. Since our patient and his family belong to a low socioeconomic background, it is important to recognize that all necessary surgical supplies, including catheter and urine bags, impose a significant financial constraint on them.

To assess the status of the QOL post-operatively, a 12-item Short-Form Health Survey (SF-12), covering the basic domains of health outcomes, including physical and social functioning, was used [7]. The questionnaire was originally produced in the English language and was later translated to Urdu to ensure proper interpretation and response of the patient. Our patient had a physical component score (PCS-12) of 54 and a mental component score (MCS-12) of 41 (Table 1). 

**Table 1:** 12-item Short-Form Health Survey (SF-12) Physical Component Score (PCS): 54 Mental Component Score (MCS): 41

Question		Response of the patient
In general, would you say your health is?	Excellent	
	Very good	
	Good	Good
	Fair	
	Poor	
Does your health now limit you in moderate activities, such as moving a table, pushing a vacuum cleaner, bowling or playing golf?	Yes, limited a lot	
	Yes, limited a little	
	No, not limited at all	No, not limited at all
Does your health now limit you in climbing several flights of stairs?	Yes, limited a lot	
	Yes, limited a little	
	No, not limited at all	No, not limited at all
During the past 4 weeks, were you able to accomplish less than you would like with your work or other regular daily activities as a result of your physical health?	Yes	
	No	No
During the past 4 weeks, were you limited in the kind of work or other regular daily activities as a result of your physical health?	Yes	
	No	No
During the past 4 weeks, were you able to accomplish less than you would like with your work or other regular daily activities as a result of any emotional problems (such as feeling depressed or anxious)?	Yes	
	No	No
During the past 4 weeks, were you unable to do work or other activities as carefully as usual as a result of any emotional problems (such as feeling depressed or anxious)?	Yes	
	No	No
During the past 4 weeks, how much did pain interfere with your normal work (including both work outside the home and housework)?	Not at all	Not at all
	A little bit	
	Moderately	
	Quite a bit	
	Extremely	
Have you felt calm and peaceful?	All the time	
	Most of the time	
	A good bit of the time	
	Some of the time	Some of the time
	A little bit of the time	
	None of the time	
Did you have a lot of energy?	All the time	
	Most of the time	
	A good bit of the time	
	Some of the time	Some of the time
	A little bit of the time	
	None of the time	
Have you felt downhearted and blue?	All the time	
	Most of the time	
	A good bit of the time	
	Some of the time	
	A little bit of the time	A little bit of the time
	None of the time	
During the past 4 weeks, how much of the time has your physical health or emotional problems interfered with your social activities (like visiting with friends, relatives, etc.)?	All the time	
	Most of the time	Most of the time
	A good bit of the time	
	Some of the time	
	A little bit of the time	
	None of the time	

The International Index of Erectile Function (IIEF) for men was used to evaluate the sexual function of our patient. However, we were unable to calculate stratified scores from this, as the patient has not yet attempted sexual intercourse. The degree of continence and prostatic function, using validated questionnaires such as International Consultation on Incontinence Questionnaire (ICIQ) and International Prostate Symptom Score (IPSS), respectively, could not be determined as our patient urinates through the stoma only. We have managed to conduct repeated psycho-sexual counseling by therapists and fertility experts to ensure that our patient receives the best possible standard of effective care. These sessions usually aim to reinforce coping skills, identify and challenge maladaptive thoughts and overall address and tackle any internalizing symptoms of the patient. Comprehensive discussions regarding urine catheterization and left kidney function have also been arranged regularly to avoid any long-term somatic complications. 

## Discussion

EEC forms a group of relatively rare congenital disorders that have posed a significant challenge to pediatric surgeons and urologists worldwide. The optimal approach towards managing such patients should also focus on dealing with the psycho-social and sexual aspects of this condition [8]. Since early mortality is no longer a concern for these patients, it is now increasingly important to recognize how reconstructive procedures and the associated congenital defect can affect the long-term QOL, establishing any necessary room for improvement in the clinical management of this disorder [9]. The management of EEC patients belonging to a low socioeconomic status from a lower-middle income country can be challenging. Of the total health care expenditure in Pakistan, 70% is out of pocket, which almost makes it less likely for patients in the lower income sectors to seek medical care and long-term follow-up. Parents with bladder exstrophy have about a 1/70 chance of giving birth to a child with exstrophy. Likewise, if a family has one child with exstrophy, they have a 1/100 chance of having another child with it. Antenatal screening for chronic cases such as EEC can drastically minimize the number of presenting cases. A literature search revealed several conditions associated with this disorder (Table 2).

**Table 2:** EEC-associated complications EEC=exstrophy-epispadias complex

	Complications
Urological defects in ~33% of patients	Stenosis/obstruction at the ureteropelvic junction
	Vesicoureteral reflux
	Ectopic kidney
	Horseshoe kidney
	Renal agenesis/dysplasia
	Ureterocele
	Ureteral ectopy
	Megaureter
Gastrointestinal defects in ~32% of patients	Omphalocele
	Umblical hernia
	Divergent distal rectus abdominis muscles
	Gastrointestinal malrotation
	Short bowel syndrome
	Duodenal atresia
Spinal/neurological defects in ~7% of patients	Neural tube defects
	Vertebral anomalies
	Tethered cord
	Dysraphism
	Myleodysplasia/myleomeningocele

Surgical management

Most patients with EEC often require pelvic osteotomies and lengthy lower extremity immobilizations to allow for adequate deepening of the pelvic cavity before the bladder can be repositioned inside; osteotomy has a complication rate of 4%, with 50% of these being neurological complications [10]. Urinary continence and the ability to void efficiently determine the QOL to a great extent in these patients, with permanent urinary diversion continuing to remain an optimum therapeutic option. Urinary continence was a major determinant of our patient’s QOL in his initial childhood as he wore diapers all the time. This can affect a child in various ways, impairing one’s physical as well as social life. A 10-year follow-up study from Germany revealed a continence rate of 95% in a group of 128 EEC patients who had undergone Mainz uretero-sigmoidostomy [11]. Continence rates following Mitrofanoff procedures have been reported to range from 79% to 100% in 23 children, as shown by Liard et al. [12]. Alternative conduits such as ureter or gastric tubes also provide good results, especially when the appendix is not available or unsuitable; however, risks of stomal leakage still exist regardless of the type of channel used [12]. 

Stomal stenosis and bladder calculi continue to be common complications routinely encountered by physicians after this procedure [5,12]. Renal calculi were observed in our patient as well. Several other techniques have been introduced in the surgical fraternity to manage EEC. Kelly’s repair involves the radical mobilization of the internal and external sphincter muscles of the bladder and urethra to achieve continence; a study done in Australia reported a 75% success rate in managing patients with this procedure [13]. Mitchell further demonstrated the single-stage concept of bladder neck reconstruction along with penile disassembly, in this way correcting the epispadias and exstrophy simultaneously, though a study done in India revealed that 44% of the patients developed penopubic fistulas [14]. The post-operative outcomes of conduit channels follow an interesting sequence of events. In a previous study, Leslie et al. reported that 65% of their patients experienced incontinence in the first three years after the initial operation followed by a smooth and fairly complication-free period later [15]. Complications related to conduits can occur later in life, possibly due to various predisposing factors such as the constant wear and tear of the channel overtime, and associated anatomical changes (particularly weight changes) [15].

To achieve suitable continence rates, concomitant bladder augmentation is usually performed alongside a Mitrofanoff procedure, especially when there are underlying congenital bladder anomalies; a study at a tertiary care hospital in Pakistan reported successful outcomes [6]. However, complications such as UTI and bladder calculus formation continue to occur even with this approach, probably due to concomitant intestinal augmentation and lack of clean catheterization techniques [5].

Psychological outcomes

Many studies have indicated that exstrophy has a significant impact on psycho-sexual functioning in patients along with anxiety and mood disorders. Following surgery, our patient also suffered from depression from time to time. This is in line with a previous study on EEC patients that revealed that internalizing symptoms (including depression, anxiety and somatization) and adaptive skills (including social skills, leadership and activities of daily living) uniformly worsened with the increasing developmental age [16]. It is interesting to note that previously only factors associated with continence predicted poor psychological functioning; however, it is the combination of normal developmental transformations and the medical complications of EEC that may contribute to increased vulnerability for psychological distress [16].

Sexual outcomes/QOL

The sexual deficits are known to be more pronounced in men as compared to women as various studies concluded that affected male patients were dissatisfied with their genital appearance and size, and preferred to maintain superficial contact with the opposite gender [17]. Patients are fearful of revealing their condition and consequently being rejected by their partner, thus lowering their self-esteem [17]. Likewise, a mean stratified score of 19.9 in the domain of erectile function was determined in a cohort of 15 patients with bladder exstrophy in the United Kingdom, thus indicating mild erectile dysfunction [9]. For QOL, a study on Dutch patients with EEC evaluated the SF-12 and reported that male participants had mental and physical scores that were comparable to the general population; differences were seen in the PCS-12 in women [18]. The trends of fertility rates in these patients also vary between both genders. A review by Woodhouse showed that paternity was twice as preserved in patients who had undergone urinary diversions as compared to those who underwent genital reconstruction [19]. Overall, a normal sperm count has been reported in 16%-63% of patients with EEC, though the volume of ejaculate has been low (mean<1.6 ml) [17,19]. However, most of the females bore normal fertility with successful pregnancies [20]. All women should be adequately counseled during pregnancy and advised elective caesarean section to avoid any risk of injury to the reconstructed urinary tract.

Our case history and review of the literature provide the most comprehensive and concise report on the treatment, complications and the QOL of patients with EEC. Data on the QOL and follow-up into post-pubertal outcomes of repair procedures are insubstantial in the current literature and should be considered in the future.

## Conclusions

In the past, EEC was a devastating birth defect, but the recent advances in bladder augmentation, pelvic osteotomies and immobilization have led to a drastic improvement in urinary continence and renal function. Nonetheless, both psychological support and psycho-sexual counseling should be provided from an earlier age to avoid any long-term personality impairments. Through our case, we highlight that patients who present in a lower socioeconomic setting from the less developed parts of the world can have a normal QOL by means of a long-term holistic multidisciplinary management approach. We have been successful in conducting effective psycho-sexual counseling sessions to ensure that the physical, emotional and social well-being of our patient is consistently evaluated and managed accordingly. 
